# Evaluating outlier loci and their effect on the identification of pedigree errors

**DOI:** 10.1186/1471-2156-6-S1-S155

**Published:** 2005-12-30

**Authors:** Ke-Sheng Wang, Michelle Liu, Andrew D Paterson

**Affiliations:** 1 Program in Genetics and Genomic Biology, The Hospital for Sick Children, 555 University Avenue, Toronto, Ontario M5G 1X8, Canada; 2 Department of Public Health Sciences, University of Toronto, 12 Queen's Park Crescent West, Toronto, Ontario, Canada

## Abstract

Homozygosity outlier loci, which show patterns of variation that are extremely divergent from the rest of the genome, can be evaluated by comparison of the homozygosity under Hardy-Weinberg proportions (the sum of the squares of allele frequencies) with the expected homozygosity under neutrality. Such outlier loci are potentially under selection (balancing selection or directional selection) when genome-wide effects (such as bottleneck and rapid population growth) are excluded. Outlier loci show skewed allele frequencies with respect to neutrality and may therefore affect the identification of pedigree errors. However, choosing neutral markers (excluding outlier loci) for the identification of pedigree errors has been neglected thus far. Our results showed that 4.1%, 5.5%, and 1.5% of the microsatellite markers, Illumina single-nucleotide polymorphisms (SNPs), and Affymetrix SNPs, respectively, on the autosomes appear to be under balancing selection (*p *≤ 0.01) while 0.8% of the Affymetrix SNPs are consistent with directional selection. On the X-chromosome, 7.7%, 3.2%, and 0.4% of the microsatellite markers, Illumina SNPs, and Affymetrix SNPs, respectively, appear to be under balancing selection. 9.3% of Illumina SNPs and 6.7% of Affymetrix SNPs which have high minor allele frequency (≥40%) appear to be under balancing selection. Pedigree structure errors in 15 of 143 pedigrees were detected using microsatellite markers from the autosomes and/or selected SNPs from chromosomes 1 to 18 of the Illumina and/or selected SNPs from chromosomes 1 to 16 of the Affymetrix. Outlier loci did not make a major difference to the identification of pedigree errors. The Collaborative Study on the Genetics of Alcoholism data has pedigree errors and some of them may be due to sample mix up.

## Background

Pedigree errors can lead to false-positive evidence for linkage or reduced power of linkage detection. In some cases, pedigree errors can be identified through the discovery of Mendelian errors. However, if some individuals in the pedigree are untyped, then Mendelian errors may not be observed. Further, single-nucleotide polymorphisms (SNPs) are mostly diallelic markers that possess just three possible genotypes and most genotyping errors may conform to Mendelian inheritance. Therefore, the identification of pedigree errors is an important first step before linkage analysis is performed.

Luikart et al. state, "Outlier loci are genomic locations (or markers or base pairs) that show behavior or patterns of variation that are extremely divergent from the rest of the genome (locus-specific effects), as revealed by simulations or statistical tests" [1]. In this study, we evaluated homozygosity outliers through comparison of the homozygosity under Hardy-Weinberg proportions (the sum of the squares of allele frequencies) with the expected homozygosity under the neutral model (mutation and random genetic drift being the only forces acting on allele frequencies) [2]. Since Kimura first suggested that most polymorphisms are selectively neutral, testing the neutral hypothesis has been one of the prime objectives of molecular population genetics [3]. For selectively neutral loci, allele number and frequency distribution in natural populations result from an equilibrium between mutations and genetic drift. In contrast to neutral loci, outlier loci are potentially under selection (balancing or directional selection) when genome-wide effects (such as bottleneck and population rapid growth) are excluded. Outlier loci show a skewed allele frequency distribution compared to neutrality and may therefore affect the identification of pedigree errors. However, until now, choosing neutral markers (excluding outlier loci) has been neglected in the identification of pedigree errors.

The present study aimed to detect the outlier loci and identify pedigree errors with genetic data as well as to compare the SNPs with microsatellite markers in identification of pedigree errors.

## Methods

We used the Collaborative Study on the Genetics of Alcoholism (COGA) cleaned data. Departure from Hardy-Weinberg equilibrium (HWE) and the neutrality of all the markers on the autosomes were tested using 239 unrelated founders of White, non-Hispanic pedigrees and on the X-chromosome using 160 unrelated female founders of White, non-Hispanic pedigrees. HWE was tested for each locus using Fisher's exact test implemented in GENEPOP v.3.4 [4,5]. Outlier loci were detected by the Ewens-Watterson homozygosity test using PYPOP v.0.5.2 [6,7]. The homozygosity statistic F (F = ∑ p_i_^2^) under HWE, is the sum of squared allele frequencies and therefore depends on the distribution of allele frequency. This F is compared to the expected homozygosity under neutrality (random neutral mutations and genetic drift), which can be obtained by simulation (dependent on the number of alleles and individuals in the population sample) [8]. In fact, this statistic tests the observed allele frequency spectrum with the expected allele frequency spectrum under the neutral model. An excess of rare alleles (homozygosity excess) indicates directional selection while an excess of intermediate frequency alleles (homozygosity deficiency) indicates balancing selection. It should be noted that homozygosity excess is not an excess of homozygotes, which refers to deviation form Hardy-Weinberg proportions. When detecting selection, we used software QVALUE [9,10] to calculate the false discovery rate (FDR) [11].

When detecting pedigree errors, in order to reduce the influence of differences in marker allele frequencies between different ethnic groups, we separated the 143 pedigrees into two subgroups: subgroup 1 includes 21 pedigrees in which a majority of individuals have self-reported their ethnicity to be 'Black' (both Hispanic and non-Hispanic) while subgroup 2 consists of 122 pedigrees which have a majority of individuals who self-reported to be 'White' (both Hispanic and non-Hispanic). For Illumina and Affymetrix datasets, we chose SNPs based on two criteria: 1) others have argued that a fraction of the available SNPs would be enough to detect pedigree errors [12,13], and 2) we did not use closely linked markers by choosing SNPs at specific intervals because the IBD (identity-by-descent) status of relative pairs for tightly linked markers is highly correlated [12]. Consequently, we used 437 SNPs selected from chromosomes 1 to 18 from the Illumina dataset which are separated by about 7 cM, and 800 SNPs selected from chromosomes 1 to 16 from the Affymetrix dataset, which are separated by about 3 cM as well as all the microsatellite markers on the autosomes. This is why the initial SNPs chosen for the identification of pedigree errors included both outlier loci and neutral loci. The outlier loci were then removed based on outlier loci detection when only neutral loci were used. PREST v.3.0 [14], which computes the conditional expected identity by descent (EIBD), adjusted identity by state (AIBS) and IBS statistics and performs the corresponding hypothesis tests for relationship misspecification [15] was used to identify pedigree errors. Then, we used ALTERTEST [14] to calculate the EIBD, AIBS, and IBS tests on the problematic relative pairs identified using PREST. As previously reported, the EIBD, AIBS, and IBS would be expected to be robust with a low rate of genotyping error in detection of pedigree errors [15]. In addition, as stated in PREST documentation, the parent-offspring relationship is a special case in identification of pedigree errors because EIBD test does not apply for parent-offspring relationships and both the AIBS and IBS tests have low power. In such cases, the likelihood-based parentage analysis software CERVUS v.2.0 [16] was used to infer the parentage of ambiguous individuals when a series of candidate parents are available [17].

## Results

On the autosomes, the proportion of outlier loci under balancing selection (*p *≤ 0.01) ranged from 1.5 to 5.5% for microsatellite markers and two SNP datasets. 0.8% of Affymetrix SNPs may be subject to directional selection (Table 1). On the X-chromosome, a lower proportion of outlier loci were detected under balancing selection but a higher proportion of outlier loci under directional selection were present in the Affymetrix SNPs. Further analysis showed that the distributions of minor allele frequencies (MAFs) in two SNP datasets are dramatically different (Table 2). For the Illumina dataset, the distribution is skewed to higher MAF, with more than half of them with MAF ≥ 40%. On the contrary, the Affymetrix dataset revealed more even MAF distribution. Comparing the proportion of loci under balancing selection with MAF < 50%, the Illumina dataset revealed a higher proportion of loci under balancing selection than Affymetrix. Furthermore, hitchhiking was observed for some loci (data not shown). When detecting selection on the autosomes, we used FDR. Based on the QVALUE, when the *p*-value cutoff is 0.01, 13 out of 315 microsatellite markers, 255 out of 4,596 Illumina SNPs, and 167 out of 10,810 Affymetrix SNPs were significant, with FDRs of 0.09, 0.08, and 0.22, respectively.

For the identification of pedigree errors, we used the significance level of α = 0.0001 for EIBD, AIBS, and IBS statistics to reduce false-positive results. Using PREST, we detected errors in 15 of 143 pedigrees (Table 3). In order to compare differences between the SNPs and microsatellite markers as well as between using all markers and just neutral markers, we list only one individual pair for each problematic individual. For pedigree 10, individual 397 is clearly unrelated to other individuals in that pedigree using both SNPs and microsatellite markers. However, some pedigree errors can be complex, e.g., individual 832 in pedigree 9 showed unclear relationship with other individuals for both SNP datasets but no problems with microsatellites. Furthermore, parentage analysis showed that individual 708 in pedigree 81 is the mother of 285. Moreover, we detected errors in 6 pedigrees (2, 16, 29, 30, 71, and 131) only with Affymetrix SNPs. Finally, we found that some unrelated individuals within the same family are likely in fact to be related. Compared with all markers, the *p*-values of EIBD and AIBS using neutral markers (without outlier loci) did not change much both for the SNPs and microsatellite markers (Table 3).

## Discussion

Outlier loci are likely to be fairly common in the human genome. There are several possible reasons for outlier loci, of which selection may be one of the most important. For instance, genome-wide factors such as a bottleneck can cause homozygosity deficiency and rapid population growth can cause homozygosity excess, but selection/selective sweep acts on specific loci. From our results, the proportions of outlier loci are relatively low in the three datasets, which indicate genome-wide factors may have minor effects on outlier loci. On the contrary, specific loci may fall in or near genes and be subjected to selection. Markers in linkage disequilibrium with such loci may also behave as non-neutral outliers. From the results of PYPOP, most of the outlier loci for the SNPs and microsatellite markers showed homozygosity deficits while a low proportion of the Affymetrix SNPs were consistent with homozygosity excess. The differences between the proportions of outlier loci for the two SNP datasets (Table 1) may be due to the differences of distribution of MAFs (Table 2). The Illumina dataset has much fewer loci with low MAF (<20%) than the Affymetrix dataset. Some of the outlier loci are located within known genes, e.g., the SNP tsc0060190 is located within gene GLO1, which is about 4 Mb centromeric to HLA on chromosome 6. This SNP has low MAF (0.4%) and is consistent with direction selection (observed F = 0.99 and expected F = 0.84,*p *< 0.00001). Second, genotyping errors may be another cause of outlier loci. Under the assumption that deviation from HWE in founders may be indicative of genotyping errors, we found 11 of 344 Affymetrix SNPs which departed from HWE (*p *≤ 0.01) appeared to be under selection (*p *≤ 0.01). However, none of the 40 Illumina SNPs that deviated from HWE appeared to be under selection. Therefore, the majority of SNPs under selection are unlikely due to genotyping errors. We also found that the deviations from HWE of these 11 Affymetrix SNPs were not due to missing data in the founders.

## Conclusion

Most of the outlier loci may be under balancing selection but a small number of them appear to be under directional selection. SNPs with high MAFs are more likely to be subject to balancing selection than SNPs with low MAFs, which has implications for the selection of SNPs in future studies. Furthermore, analysis of 437 Illumina SNPs in a 7-cM scan and 800 Affemetrix SNPs in a 3-cM scan showed similar results to using 315 microsatellite markers for detecting the pedigree errors. Meanwhile, the influence of outlier loci on identification of pedigree errors appears to be minor. Moreover, several pedigrees showed different results using SNPs and microsatellite markers, which may be due to sample mix-up. In addition, PREST has been used in COGA data – Genetic Analysis Workshop (GAW) 11 [15] where 26 of 949 pairs (from 11 pedigrees) were significant (*p *< 0.001). However, it is not clear whether the GAW 11 COGA pedigrees overlap with the GAW14 COGA pedigrees.

## Abbreviations

AIBS: Adjusted identity by state

COGA: Collaborative Study on the Genetics of Alcoholism

EIBD: Expected identity by descent

FDR: False discovery rate

GAW: Genetic Analysis Workshop

HWE: Hardy-Weinberg equilibrium

IBD: Identity by descent

MAF: Minor allele frequencies

SNP: Single-nucleotide polymorphism

## Authors' contributions

KSW performed the statistical analysis and drafted the manuscript. ML and ADP participated in the design of the study and interpretation of the results. All authors have read and approved the final version of this manuscript.

## Figures and Tables

**Table 1:** Proportion of outlier loci at different significance levels

Marker type	Chromosome	1% level (BS)^a^	0.1% level (BS)	0.01% level (BS)	0.01% level (DS)^b^
Microsatellite	Autosome	4.1%	1.3%	0.6%	0%
	X-chromosome	7.7%	0%	0%	0%
Illumina	Autosome	5.5%	0.9%	0%	0%
SNPs	X-chromosome	3.2%	0%	0%	0%
Affymetrix	Autosome	1.5%	0.2%	0%	0.8%
SNPs	X-chromosome	0.4%	0%	0%	2%

^a ^BS, balancing selection

^b ^DS, directional selection

**Table 2:** Proportion of outlier loci within different minor allele frequency (MAF) intervals

MAF interval (%)	Proportion of all SNPs		Loci under balancing selection (1% level)		Loci under directional selection (0.01% level)	
			
	Illumina	Affymetrix	Illumina	Affymetrix	Illumina	Affymetrix
0 – 9.99	0.1%	12.5%	0%	0%	0%	6.4%
10 – 19.99	3.4%	19.5%	0%	0%	0%	0.1%
20 – 29.99	12.4%	22.5%	0.3%	0.1%	0%	0.1%
30 – 39.99	26.7%	23.3%	0.6%	0%	0%	0%
40 – 49.99	57.4%	22.3%	9.3%	6.7%	0%	0%

**Table 3:** Pedigree errors identified in the COGA data

				With outlier loci						Without outlier loci					
					
Ped^a^	ID in error^b^	ID of relative^c^	Description	SNPs (Illumina)		SNPs (Affymetrix)		Microsatellite		SNPs (Illumina)		SNPs (Affymetrix)		Microsatellite	
									
				P0^d^	AIBS^e^	P0	AIBS	P0	AIBS	P0	AIBS	P0	AIBS	P0	AIBS
2	1540	530	Avuncular of 530	0.411	0.390	0.225	0	0.423	0.142	0.435	0.605	0.224	0	0.450	0.179
9	832	1459	full sib of 1459	0.738	0^h^	0.741	0	0.244	0.409	0.700	0	0.718	0	0.202	0.383
10	397	887	full sib of 887	0.997	0	0.593	0	0.842	0	1	0	0.583	0	0.872	0
34	1272	616^f^	half sib of 616	0.327	0.663	0.184	0	0.255	0.002	0.297	0.471	0.227	0	0.252	0.004
50	1406	313	Avuncular of 313	0.293	0.894	0.505	0.292	0.308	0	0.407	0.941	0.549	0.239	0.323	0
52	515	1246	first cousin of 1246	0.197	0	0.161	0	0.248	0	0.179	0	0.178	0	0.278	0
59	1062	1375	full sib of 1375	0.98	0	0.964	0	0.298	0.130	0.992	0	0.950	0	0.282	0.177
81	418	1060	full sib of 1060	0.497	0	0.321	0.003	0.334	0.114	0.509	0	0.364	0.007	0.318	0.176
81	285	708	parent-offspring relationship of 708	NA^g^	NA	0	0	0	0	NA	NA	0	0	0	0
126	259	505	half sib of 505	0.196	0	0.118	0	0.865	0.00008	0.153	0	0.117	0	0.857	0.00013

^a ^Ped = pedigree id

^b ^ID in error = id of individual which has problematic genetic relationships with other individuals within the pedigree

^c ^ID of relative = id of individuals which are relatives to id in error

^d ^P0 = the probability of sharing 0 alleles IBD

^e ^AIBS = two-sided *p*-value for the adjusted IBS test

^f ^individual 616 is genotyped for only 163 microsatellite markers.

^g ^NA = 708 has no genotype data

^h ^0 refers to *p*-values smaller than 0.00001
